# Enhanced Photocatalytic and Photokilling Activities of Cu-Doped TiO_2_ Nanoparticles

**DOI:** 10.3390/nano12071198

**Published:** 2022-04-03

**Authors:** Yumatorn Mingmongkol, Dang Trung Tri Trinh, Patcharaporn Phuinthiang, Duangdao Channei, Khakhanang Ratananikom, Auppatham Nakaruk, Wilawan Khanitchaidecha

**Affiliations:** 1 Department of Civil Engineering, Faculty of Engineering, Naresuan University, Phitsanulok 65000, Thailand; yumatorn_nk@hotmail.com (Y.M.); ryeoploy09@gmail.com (P.P.); 2 Centre of Excellence for Innovation and Technology for Water Treatment, Faculty of Engineering, Naresuan University, Phitsanulok 65000, Thailand; tttdang247@gmail.com (D.T.T.T.); auppathamn@nu.ac.th (A.N.); 3 Institute of Environmental Science & Technology, Tra Vinh University, Tra Vinh 87000, Vietnam; 4 Department of Chemistry, Faculty of Science, Naresuan University, Phitsanulok 65000, Thailand; duangdaoc@nu.ac.th; 5 Department of Science and Mathematics, Faculty of Science and Health Technology, Kalasin University, Kalasin 46000, Thailand; khakhanang_r@yahoo.com; 6 Department of Industrial Engineering, Faculty of Engineering, Naresuan University, Phitsanulok 65000, Thailand

**Keywords:** Cu-doped TiO_2_, hydrothermal, nanoparticles, photocatalytic, photokilling

## Abstract

In this work, metal-doped titanium dioxide (TiO_2_) was synthesised with the aim of improving photocatalytic degradation and antimicrobial activities; TiO_2_ was doped with copper (Cu) ranging from 0.1 to 1.0 wt%. The physical and chemical properties of the Cu-doped TiO_2_ nanoparticles were characterized by X-ray diffraction (XRD), transmission electron microscopy (TEM), the Brunauer–Emmett–Teller method (BET) and diffuse reflection spectroscopy (DRS). The results revealed that the anatase phase of TiO_2_ was maintained well in all the Cu-doped TiO_2_ samples. No significant difference in the particle sizes or the specific surface areas was caused by increasing Cu doping. However, the band gap decreased continuously from 3.20 eV for undoped TiO_2_ to 3.12 eV for 1.0 wt.% Cu-doped TiO_2_. In addition, the 0.1 wt.% Cu-doped TiO_2_ displayed a much greater photocatalytic degradation of methylene blue (MB) and excellent antibacterial ability for *Escherichia coli* (*E. coli*) compared to undoped TiO_2_. On the other hand, the high Cu doping levels had negative impacts on the surface charge of nanoparticles and charge transfer for OH• generation, resulting in decreasing MB degradation and *E. coli* photokilling for 1.0 wt.% Cu-doped TiO_2_.

## 1. Introduction

Over the past several decades, the use of titanium dioxide (TiO_2_, titania) has been increasing significantly due to its advantages and capabilities for various applications, including the antibacterial disinfection of surfaces, self-cleaning and self-sterilization, site remediation, domestic and industrial wastewater treatments [1,2,3]. In addition, TiO_2_ is highly chemically-stable and human-friendly [4,5], therefore, it can also be used for biomaterials, biomedical devices and food applications [6,7]. It must be noted that TiO_2_ nanoparticles are considered safe: they are used as food additives and for surface coating materials [8,9]. Their safety has been investigated using a model intestinal bacterial community, indicating that TiO_2_ does not significantly alter the human gut microbiome [9]. Theoretically, TiO_2_ has a band gap of 3.20 eV, which means the photocatalytic activity can be activated only under UV regions. Therefore, reducing the TiO_2_ band gap is one of the main goals of researchers in the field, because it can enhance the photocatalytic activity under visible light regions.

Various narrow-band-gap materials such as cerium dioxide (CeO_2_) and bismuth vanadate (BiVO_4_) are active under visible light and exhibit promising photocatalytic activity in the degradation of organic compounds. The redox potential of Ce^4+^/Ce^3+^ makes it a particularly effective photocatalyst of CeO_2_, yet the wide band gap range of 2.6 to 3.4 eV, depending on the preparation methods, limits the applications of CeO_2_, though it can adsorb a larger fraction of the solar spectrum rather than TiO_2_ [10]. Conversely, BiVO_4_ has a relatively small band gap of ~2.4 eV and its high photocatalytic activity under visible light has attracted attention to its use as a photocatalyst. However, low photocatalytic activity of BiVO_4_ has been observed due to its weak adsorption performance and poor migration of charged carriers [11].

There are several methods of reducing the band gap of TiO_2_, but one of the most well-known methods involves doping with transition metals. Many metals have been introduced for this purpose, such as Mn [12], Co [13], Zn [14] and Cu [15]. The latter has several significant advantages; for example, Cu can create multi-bands, act as inhibitor of grain growth and extend the electron-hole (e^−^, h^+^) pair recombination. In addition, an improvement in the visible light adsorption of TiO_2_ was observed after doping with Cu, resulting in the further promotion of photocatalytic efficiency [16]. Leading research has further shown that Cu-doped TiO_2_ provided superior antibacterial performance [17,18,19,20,21,22,23,24]. These studies proved that Cu-doped TiO_2_ can be active under UV-A and visible light, and also that it can kill 100% of microbes. In addition, Cu has also been used in various forms of TiO_2,_ for example, deposited on the top layer of TiO_2_ thin film [25], nanocomposite forms of CuO-TiO_2_ [26] and grafting Cu^2+^ in TiO_2_ and WO_2_ structures [27]. The well-known synthesis method of Cu-doped TiO_2_ nanoparticles is the sol-gel technique; the nanoparticles are calcined at high temperatures ranging from 500 to 700 °C [16,28], and Cu precursors, such as copper chloride (CuCl_2_) and copper sulphate (CuSO_4_), are added to the titanium solution. The precipitation method has also been used to synthesise the Cu-doped nanocrystalline TiO_2_; this method can be carried out using low-cost materials and easier manufacturing methods at industrial levels. Another attractive hydrothermal method, employed under self-produced pressures at low temperatures, can produce highly homogeneous nanoparticles with fewer contamination phases and less particle agglomeration [29,30]. Among the above-mentioned techniques, the hydrothermal method is very interesting due to its significant advantages: it makes it easy to obtain nanostructured morphology, a variation in the synthesis method can be implemented to enhance the properties of TiO_2_, and it is a feasible method for varying applications.

Furthermore, TiO_2_-based composites have been synthesised to improve material properties, photocatalytic activity and degradation of organic compounds. The graphene–TiO_2_ composite can produce a large number of pores, which increases the active photocatalytic sites and provides space for adsorption [31]. This composite also showed efficient degradation of pollutants under both visible light and sunlight [31]. Another composite of Zeolite Socony Mobil-5 (ZSM-5) and TiO_2_ displayed great surface area and mesopore volume. However, the synthesis method of the ZSM-5/TiO_2_ composite affected material properties and pollutant degradation [32].

Although the Cu-doped TiO_2_ is well-recognized for antibacterial performance, the doping ratio of Cu/Ti is still doubtful at present. The doping percentage can be found over the range of 0.1 up to 10.0 wt.%. In this work, we have succeeded in discovering the best Cu doping weight percentage that could provide the best photokilling efficiency. In addition, a one-step process of a hydrothermal method has been demonstrated in the present work for synthesising various weight percentages of Cu-doped TiO_2_ nanoparticles. These nanoparticles were also characterised in physical and chemical properties, including the structure, topology and band gap energy. In addition, the improved photocatalytic and antibacterial abilities of Cu-doped TiO_2_ nanoparticles were examined and discussed in comparison to undoped TiO_2_.

## 2. Experimental Procedure

### 2.1. Cu-Doped TiO_2_ Nanoparticle Synthesis

Undoped and Cu-doped TiO_2_ nanoparticles were synthesised using hydrothermal methods. A 4 mL volume of titanium (IV) isopropoxide (TTIP: 97% reagent grade, Sigma-Aldrich) and 20 mL of ethanol (>99% reagent grade, Merck) were mixed, and then 4 mL of deionized (DI) water was slowly added to the solution. After stirring at 200 rpm for 1 h at room temperature (~25 °C), the solution was transferred to a Teflon-lined stainless steel autoclave and heated at 180 °C for 12 h. Finally, the TiO_2_ nanoparticles were washed with DI water and dried at 100 °C for 12 h. For Cu-doped TiO_2_ nanoparticles, various weight percentages of Cu, including 0.1, 0.5 and 1.0 wt.% of the aqueous solution, were prepared by dissolving CuSO_4_·5H_2_O (>98% reagent grade, Merck) in 100 mL of DI water. The Cu solution was added to the titanium solution during the stirring. The mixed solution was transferred to a Teflon-lined stainless steel autoclave, and the same procedure as for TiO_2_ nanoparticle preparation was followed.

### 2.2. Characterisation of Nanoparticles

The structural properties and phase identification of nanoparticles were examined using X-ray diffraction (XRD: Bruker, D2 Phaser) under Cu Kα radiation (λ = 0.154 nm) and 2θ ranging from 20° to 80°. The topological properties of nanoparticles were investigated using a transmission electron microscope (TEM: JEOL, JEM-2100Plus; Japan) with an acceleration voltage of 100 kV. In addition, the specific surface area of nanoparticles (SSA) was obtained with the Brunauer–Emmett–Teller method (BET: Micromeritics, TriStar-II-3020) using nitrogen adsorption-desorption analysis at 77 K. Furthermore, the optical band gap of nanoparticles was determined using diffuse reflection spectroscopy (DRS: Shimadzu, UV-360) with an integrating sphere attachment (ISR-3100, Shimadzu; Japan). The band gap energy was calculated using a Tauc plot from the DRS spectrum [33].

### 2.3. Photocatalysis and Photokilling Examinations

Photodegradation of methylene blue (MB) was used to evaluate the photocatalytic activity of undoped and Cu-doped TiO_2_ nanoparticles. A 10^−5^ M MB concentration (~3.2 mg/L) was prepared in DI water. The undoped and Cu-doped TiO_2_ nanoparticles were dispersed in the MB solution at a 1 g/L concentration. Meanwhile, the MB removal efficiency using synthesised nanoparticles was compared to that of Degussa P25 (≥99.5% trace metals basis, Sigma-Aldrich), which is a well-known benchmark TiO_2_ with high photocatalytic efficiency. The MB solutions with nanoparticles were kept in the dark for 30 min at room temperature, and afterwards, they were radiated with UV-A (365 nm, single wavelength) with magnetic stirring at 100 rpm. The remaining MB concentration was measured with UV-VIS spectroscopy (UV-6100, Mapada; China), and the MB removal efficiency was calculated.

**Figure 1:**
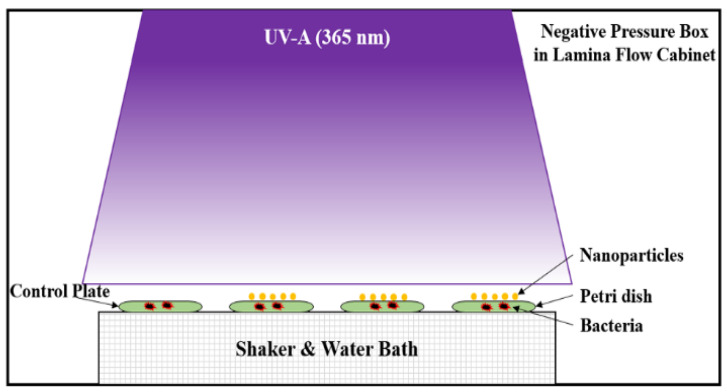
The layout of the photo-killing experimental setup.

Gram-negative bacteria, *Escherichia coli* *(E. coli)* TISTR117 (from Thailand Institute of Scientific and Technological Research), were used as indicator strains for a photokilling evaluation of nanoparticles. The indicator strain was grown in nutrient broth at 30 °C for 16–18 h, and afterwards, the bacteria culture was diluted in DI water to obtain the initial concentration of 10^7^ CFU/mL. The 10 mL bacterial suspensions were individually treated with 0.025 g of undoped TiO_2_ and 0.1 wt.%, 0.5 wt.% and 1.0 wt.% Cu-doped TiO_2_. It must be noted that the untreated sample (without nanoparticles) represented the control in this study. The treated samples and untreated sample were activated under 10 W of UV-A for 0, 30, 60, 120 and 180 min on the rotary platform. The layout and configuration of the experiment are presented in Figure 1. The visible cell growth was monitored by plating 100 μL of treated and untreated samples on the nutrient agar in chronological order and incubating them at 30 °C for 24 h. The number of visible colonies were counted and the bacteria viability was determined by plotting log of visible counts (CFU/mL) against incubation time under UV-A radiation.

## 3. Results and Discussions

**Figure 2:**
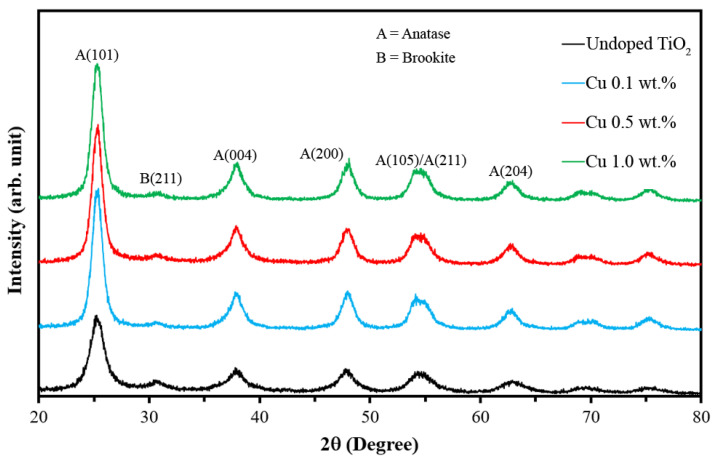
XRD patterns of Cu-doped TiO_2_ nanoparticles, Anatase: ICCD card no. 00-064-0863, Brookite: ICCD card no. 01-071-4943.

Figure 2 shows the XRD patterns of undoped and Cu-doped TiO_2_ nanoparticles. It can be seen that undoped TiO_2_ consisted of two phases: anatase, as a major phase, and brookite, as a minor phase. On the other hand, Cu-doped nanoparticles contained only the sole phase of anatase. The data also indicate that as doping increased, the degree of crystallinity also increased; the reason is that Cu is known as a promoter in the grain growth structure, so it can help to promote the anatase structure [34]. The lattice parameter data and crystallite size were calculated using XRD data, and the results are presented in Table 1. The lattice parameter data suggest that the unit cell is slightly shirked with increasing Cu doping levels. This is due to the replacement of the Ti^4+^ with the Cu^2+^. Since the ionic radius of Ti is 0.61 Å and of Cu is 0.57 Å [35], the unit cell will be compressed. The crystallite size data were also similar to the data from the TEM image (7–10 nm). Therefore, these nanoparticles form a single grain particle.

**Figure 3:**
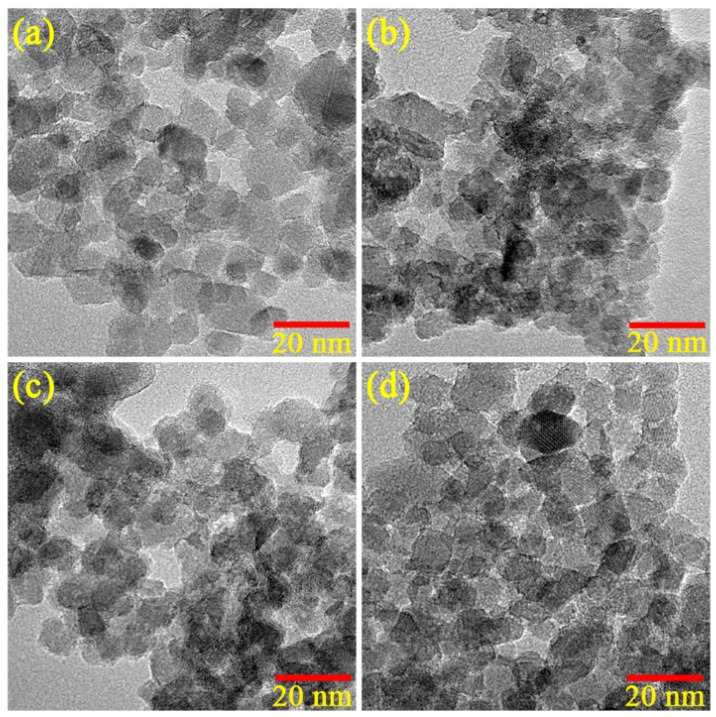
TEM images of (**a**) undoped, (**b**) 0.1 wt.%, (**c**) 0.5 wt.% and (**d**) 1.0 wt.% Cu-doped TiO_2_ nanoparticles.

TEM images of the nanoparticles are presented in Figure 3. The undoped TiO_2_ nanoparticles had consistent particle sizes of 10 nm. Meanwhile, the Cu-doped TiO_2_ nanoparticles tended to show similar particle sizes to the undoped TiO_2_ nanoparticles at 10 nm. In addition, the SSA data are given in Table 1. The data reveal that the SSA of all the samples was in the same range of 180–182 m^2^/g. Generally, the photocatalytic performance depends on three key parameters: the optical band gap, specific surface area and electron–hole recombination rate. Since the SSA of all the samples was similar, the photocatalytic performance would rely on the optical band gap and electron-hole recombination rate. Both parameters are discussed below.

**Figure 4:**
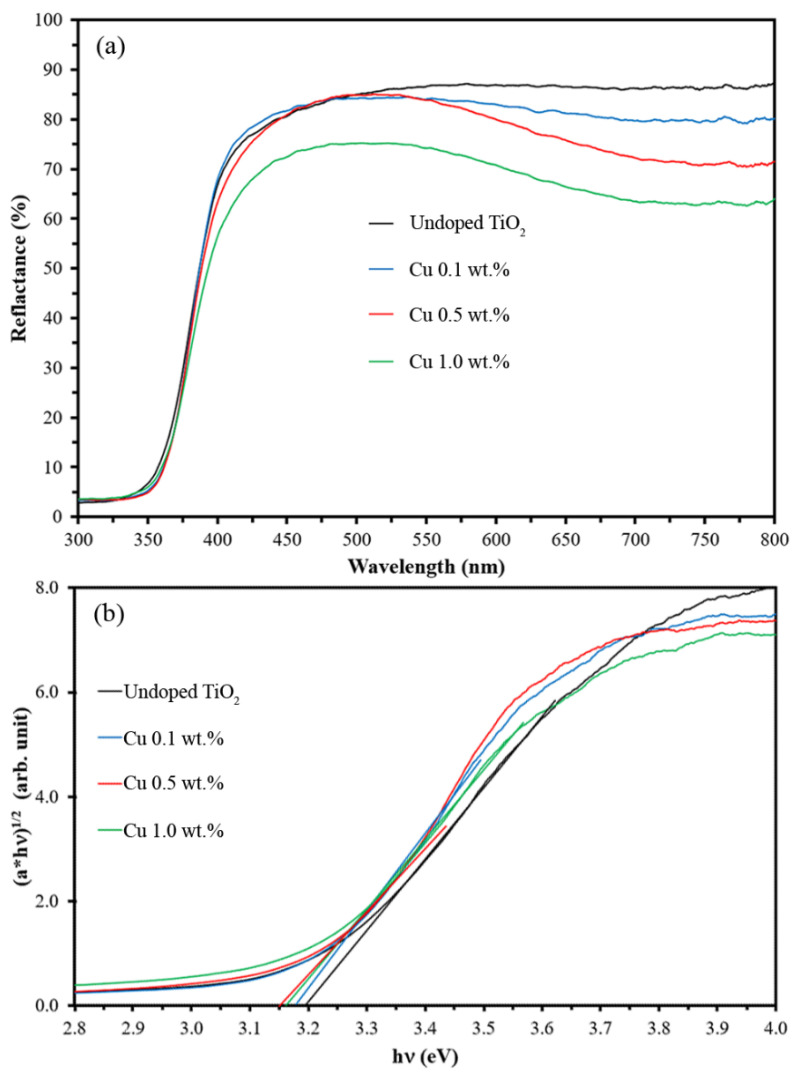
Reflection spectra (**a**) and Tauc plot (**b**) of Cu-doped TiO_2_ nanoparticles.

According to the structure of Cu-doped TiO_2_ nanoparticles, the substitution of Ti(IV) by Cu(II) was inconsistent due to the difference between ionic radii (Ti(IV) = 0.61 Å and Cu(II) = 0.57 Å) [35]. However, the smaller Cu(II) was simply replaced with the host of larger Ti(IV) in the Cu-doped TiO_2_ nanoparticles, causing the compression of the unit cell. To explain the charge balance of Cu-doped TiO_2_ on the electronic structures, band gap and band edge positions; doping elements of Cu^2+^ into TiO_2_ induces effective charges in solid state compounds. When the Cu^2+^ replaces Ti^4+^ in the TiO_2_ lattice, the system must be compensated by either cation vacancies or free electrons, or changed of valence state of Ti^4+^ ions. It is important to understand how the additional charges can affect the system and the subsequent influence on the band gap transitions. In this case, the forms of the charge-balance structures of Cu-doped TiO_2_ lead to the decreasing of the optical band gap, as showing Figure 4.

**Figure 5:**
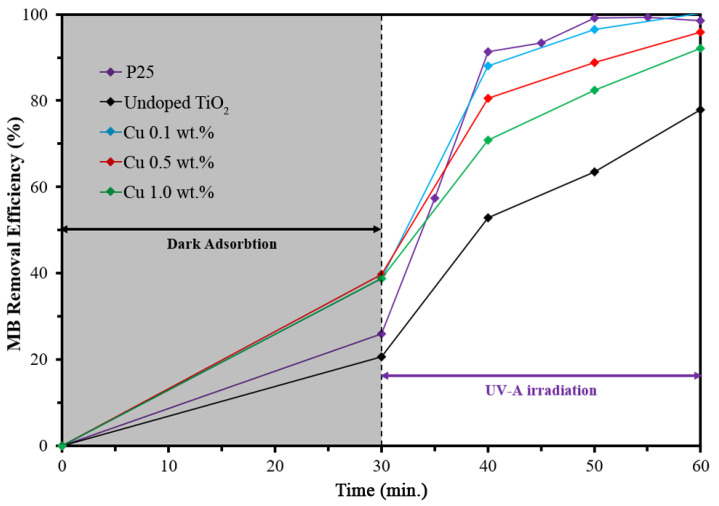
Photocatalytic activity of undoped and Cu-doped TiO_2_ nanoparticles for methylene blue (MB) degradation.

According to the Tauc plot [33], the result revealed that the band gap was reduced by increasing Cu doping levels, as shown in Figure 4. This is because the Cu^2+^ ions generated sub-bands near conduction band, resulting in the band gap reduction of Cu-doped TiO_2_. Concurrently, the sub-bands also increased the electron-hole recombination time. These two phenomena increased the performance of photocatalytic efficiency, as shown in the MB degradation test presented in Figure 5.

The photocatalytic performance of nanoparticles was evaluated by measuring the degradation of MB and the photokilling of *E. coli*. As shown in Figure 5, the results clearly indicate that Cu doping can increase the MB degradation of TiO_2_ nanoparticles. It is important to note that the undoped TiO_2_ and benchmark TiO_2_ (P25) achieved only ~20% of MB removal in the dark adsorption, whereas the Cu-doped TiO_2_ nanoparticles achieved ~40 of MB removal. Due to the similar SSA and particle sizes of undoped and Cu-doped TiO_2_, the surface charge of the Cu-doped TiO_2_ possibly caused the improvement in the MB adsorption ability. As reported in the literature [36], the pH of TiO_2_, of which the zeta potential shifted to zero (point of zero charge, P_ZC_), was approximately 6.5. The P_ZC_ tended to move towards the lower pH when the Cu doping was increased: it was approximately 6.0 for 0.15 wt.% Cu-doped TiO_2_. The pH value of the initial MB solution was measured to be 6.2, which was above the P_ZC_ of Cu-doped TiO_2_. Therefore, the surface charge of nanoparticles was negative. The cationic MB dye with a positive charge favoured the electrostatic interaction with the negatively charge surface of Cu-doped TiO_2_ nanoparticles. This phenomenon leaded to the increase in adsorption ability in the dark as well as the MB degradation rate under UV-A irradiation.

Under UV-A irradiation, the 0.1 wt.% Cu-doped TiO_2_ nanoparticles demonstrated very high performance, similar to that of P25, in terms of MB degradation. Since the Cu atoms generated sub-bands in the Cu-doped TiO_2_ structure, this may have caused the decrease in the optical band gap [20] in this work (as shown in Table 1 and Figure 4). The sub-bands can trap the exited electron from the exited state, resulting in the slow electron-hole recombination [18] and increasing the chances of hydroxyl radical (HO•) generation [37]. Furthermore, hydrogen peroxide (H_2_O_2_) is the intermediate pathway of the photocatalytic process and can react with Cu^2+^ ions and generate a Fenton-like reaction to degrade MB. The hybrid process of the Fenton-like reaction and photocatalysis of using 0.1 wt.% Cu-doped TiO_2_ nanoparticles significantly improves the MB degradation.

On the other hand, the ion doping also acts as an impurity that creates structural defects, which negatively affects the photocatalytic performance [21]. Since the defects can trap and/or quench the excited electron and hole, the high Cu content of Cu-doped TiO_2_ nanoparticles makes it the recombination centre for photogenerated electron-hole pairs [38,39]. It is also interesting to consider that at the high dopant concentration, the charge trapping is high and the charge carrier pairs may recombine though quantum tunnelling [40]. In this work, the MB removal efficiency decreased to 95.83% and 92.17% for 0.5 wt.% and 1.0 wt.% Cu-doped TiO_2_, respectively.

**Figure 6:**
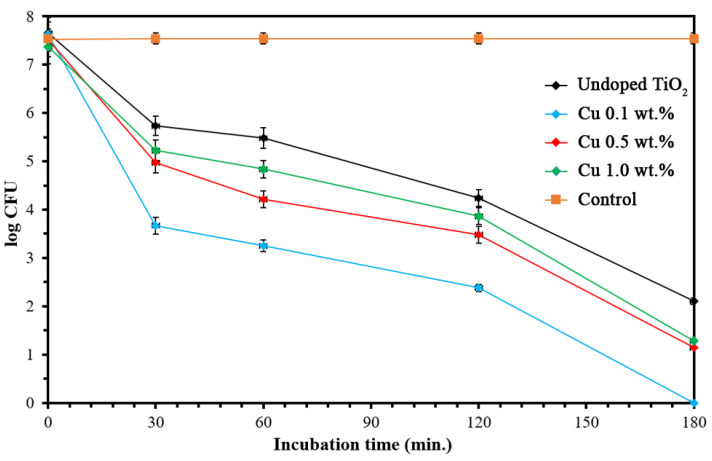
Antibacterial activity of undoped and Cu-doped TiO_2_ nanoparticles.

**Figure 7:**
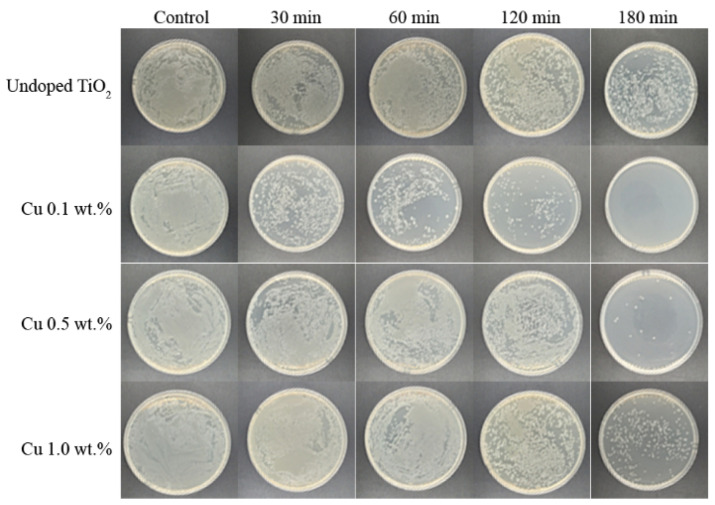
Photo of visible *E. coli* colony after UV-A radiation.

The photocatalytic performance of nanoparticles was clarified by the photokilling activity of undoped and Cu-doped TiO_2_ nanoparticles against *E. coli*. Figure 6 and Figure 7 clearly present that in the control sample (the bacterial culture without nanoparticles), the UV-A irradiation did not cause any bacteria death, as shown by the steady growth curve over the incubation time. A decreasing curve of the visible bacteria count was observed in the bacterial culture with either undoped or Cu-doped TiO_2_ nanoparticles, meaning that both undoped and Cu-doped TiO_2_ nanoparticles showed antibacterial and bactericidal activities. The indicator strain was completely killed in 180 min for the 0.1 wt.% Cu-doped TiO_2_ nanoparticles, whereas the undoped TiO_2_ showed a slight inhibition of bacterial growth. In addition, the higher Cu doping of 0.5 wt.% and 1.0 wt.% Cu-doped TiO_2_ nanoparticles achieved lower *E. coli* photokilling than the 0.1 wt.% Cu-doped TiO_2_ nanoparticles. Furthermore, some visible bacteria growth was observed after 180 min of UV-A irradiation. It can therefore be concluded that the photokilling activity of TiO_2_ nanoparticles can be enhanced by doping Cu into TiO_2_ nanoparticles; however, its photokilling activity did not follow in a dose-dependent manner. The high Cu doping levels had negative impacts on photocatalytic performance, including MB degradation and photokilling activity, as explained above. It has to be noted that Cu-doped TiO_2_ can perform the photokilling activity under dark conditions with less efficiency compared to under UV irradiation [41,42,43].

Cu-doped TiO_2_ can perform some photokilling in dark conditions. The antibacterial property of CuO, Cu and Cu-doped TiO_2_ demonstrates the high performance of killing bacteria without any radiation. The UV radiation enhanced the electron transfer and the ROS generation against bacteria cells, resulting in inactivation of bacteria cells. However, it needs to be noted that the antibacterial activity of CuO-doped TiO_2_ nanomaterial was not investigated in our study. The cooperative effect of CuO and TiOs in CuO-doped TiO_2_ nanoparticles was our main interest.

According to this work, the 0.1 wt.%. Cu-doped TiO_2_ nanoparticles showed the best antibacterial activity. The antibacterial mechanism of TiO_2_ has been explained in previous studies [25,44,45,46]. This was mainly in relation to the generation of strong reactive oxygen species (ROS) (i.e., HO•, O_2_^−^). These oxygen species attached to the cell membrane and activated the peroxidation of the polyunsaturated phospholipid component of the cell membrane. The change in cell integrity lead to the leakage of cell components and eventual cell death. In addition, Cu doping into TiO_2_ can enhance antibacterial activity because Cu itself is antibacterial due to the Fenton-like reaction. Furthermore, the combination of Cu and TiO_2_ reduced the charge carrier recombination, resulting in an increased chance of reaction with water and oxygen to generate ROS, which were responsible for destroying the bacterial cells. Furthermore, Ansari et al. discovered that the TiO_2_ nanofibres were more active against Gram-negative cells than Gram-positive cells. Since *E. coli* is a Gram-negative bacterium, other Gram-positive bacteria (e.g., *S. aureus*) should be further tested to demonstrate the extent of the photokilling performance of 0.1 %wt. Cu-doped TiO_2_ [47].

## 4. Conclusions

This work illustrated an approach to improve the photocatalytic activity of TiO_2_ by doping with Cu^2+^ ranging from 0.1 to 1.0 wt%. The coupling of nanoparticles between TiO_2_ and Cu led to a decrease in the band gap from 3.20 eV to 3.12 eV; however, the particle sizes and specific surface areas of all the Cu-doped TiO_2_ samples were similar to the undoped TiO_2_ at approximately 10 nm and 180–182 m^2^/g, respectively. The enhanced photocatalytic activity of Cu-doped TiO_2_ nanoparticles was verified by MB degradation and *E. coli* photokilling. The greatest photocatalytic activity was observed in the 0.1 wt.% Cu-doped TiO_2_ nanoparticles, which showed approximately 100% MB degradation and *E. coli* photokilling. However, the fraction of Cu doping significantly impacted the surface charge together with charge transfer for HO• generation, resulting in a decrease in the photocatalytic activity of 1.0 wt.% Cu-doped TiO_2_ nanoparticles. Therefore, the optimal fraction of metal doping was an important factor for enhancing the photocatalytic activity of TiO_2_.

## Figures and Tables

**Table 1:** Summary of analytical data.

Parameters	Samples			
	Undoped TiO_2_	0.1 wt.% Cu-Doped	0.5 wt.% Cu-Doped	1.0 wt.% Cu-Doped
Phase	Anatase			
Lattice parameter (Å)	a = 3.786 c = 9.503	a = 3.787 c = 9.496	a = 3.787 c = 9.488	a = 3.788 c = 9.484
Crystallite Size (nm)	7.73	7.86	7.84	7.85
Particle size (nm)	~10			
Specific surface area (m^2^/g)	181.34	180.20	182.32	180.00
Band gap (eV)	3.20	3.15	3.10	3.12
MB removal efficiency, after 60 min of irradiation time (%)	77.86%	100%	95.83%	92.17%

## Data Availability

Not applicable.

## References

[1] Kumaravel V., Nair K.M., Mathew S., Bartlett J., Kennedy J.E., Manning H.G., Whelan B.J., Leyland N.S., Pillai S.C. (2021). Antimicrobial TiO_2_ nanocomposite coatings for surfaces, dental and orthopaedic implants. Chem. Eng. J..

[2] Liu H., Feng Y., Shao J., Chen Y., Wang Z.L., Li H., Chen X., Bian Z. (2020). Self-cleaning triboelectric nanogenerator based on TiO_2_ photocatalysis. Nano Energy.

[3] Threrujirapapong T., Khanitchaidecha W., Nakaruk A. (2017). Treatment of high organic carbon industrial wastewater using photocatalysis process. Environ. Nanotechnol. Monit. Manag..

[4] Kaur N., Mahajan A., Bhullar V., Singh D.P., Saxena V., Debnath A.K., Aswal D.K., Devi D., Singh F., Chopra S. (2020). Ag ion implanted TiO_2_ photoanodes for fabrication of highly efficient and economical plasmonic dye sensitized solar cells. Chem. Phys. Lett..

[5] Geppert M., Schwarz A., Stangassinger L.M., Wenger S., Wienerroither L.M., Ess S., Duschl A., Himly M. (2020). Interactions of TiO_2_ Nanoparticles with Ingredients from Modern Lifestyle Products and Their Effects on Human Skin Cells. Chem. Res. Toxicol..

[6] Cheung K.H., Pabbruwe M.B., Chen W.-F., Koshy P., Sorrell C.C. (2021). Thermodynamic and microstructural analyses of photocatalytic TiO_2_ from the anodization of biomedical-grade Ti6Al4V in phosphoric acid or sulfuric acid. Ceram. Int..

[7] Kordjazi Z., Ajji A. (2021). Development of TiO_2_ catalyzed HTPB based oxygen scavenging films for food packaging applications. Food Control.

[8] Yemmireddy V.K., Hung Y.-C. (2017). Using Photocatalyst Metal Oxides as Antimicrobial Surface Coatings to Ensure Food Safety—Opportunities and Challenges. Compr. Rev. Food Sci. Food Saf..

[9] Dudefoi W., Moniz K., Allen-Vercoe E., Ropers M.-H., Walker V.K. (2017). Impact of food grade and nano-TiO_2_ particles on a human intestinal community. Food Chem. Toxicol..

[10] Kusmierek E. (2020). A CeO_2_ Semiconductor as a Photocatalytic and Photoelectrocatalytic Material for the Remediation of Pollutants in Industrial Wastewater: A Review. Catalysts.

[11] Trinh D.T.T., Channei D., Nakaruk A., Khanitchaidecha W. (2021). New insight into the photocatalytic degradation of organic pollutant over BiVO_4_/SiO_2_/GO nanocomposite. Sci. Rep..

[12] Prashad Ojha D., Babu Poudel M., Joo Kim H. (2020). Investigation of electrochemical performance of a high surface area mesoporous Mn doped TiO_2_ nanoparticle for a supercapacitor. Mater. Lett..

[13] Lim J., Yang Y., Hoffmann M.R. (2019). Activation of Peroxymonosulfate by Oxygen Vacancies-Enriched Cobalt-Doped Black TiO_2_ Nanotubes for the Removal of Organic Pollutants. Environ. Sci. Technol..

[14] Alotaibi A.M., Promdet P., Hwang G.B., Li J., Nair S.P., Sathasivam S., Kafizas A., Carmalt C.J., Parkin I.P. (2021). Zn and N Codoped TiO_2_ Thin Films: Photocatalytic and Bactericidal Activity. ACS Appl. Mater. Interfaces.

[15] Moon E.W., Lee H.-W., Rok J.H., Ha J.-H. (2020). Photocatalytic inactivation of viral particles of human norovirus by Cu-doped TiO_2_ non-woven fabric under UVA-LED wavelengths. Sci. Total Environ..

[16] Mathew S., Ganguly P., Rhatigan S., Kumaravel V., Byrne C., Hinder S.J., Bartlett J., Nolan M., Pillai S.C. (2018). Cu-Doped TiO_2_: Visible Light Assisted Photocatalytic Antimicrobial Activity. Appl. Sci..

[17] Karunakaran C., Abiramasundari G., Gomathisankar P., Manikandan G., Anandi V. (2010). Cu-doped TiO_2_ nanoparticles for photocatalytic disinfection of bacteria under visible light. J. Colloid Interface Sci..

[18] Yang X.-j., Wang S., Sun H.-m., Wang X.-b., Lian J.-S. (2015). Preparation and photocatalytic performance of Cu-doped TiO_2_ nanoparticles. Trans. Nonferrous Met. Soc. China.

[19] Wu B., Huang R., Sahu M., Feng X., Biswas P., Tang Y.J. (2010). Bacterial responses to Cu-doped TiO_2_ nanoparticles. Sci. Total Environ..

[20] Aguilar T., Navas J., Alcántara R., Fernández-Lorenzo C., Gallardo J.J., Blanco G., Martín-Calleja J. (2013). A route for the synthesis of Cu-doped TiO_2_ nanoparticles with a very low band gap. Chem. Phys. Lett..

[21] Choudhury B., Dey M., Choudhury A. (2013). Defect generation, d-d transition, and band gap reduction in Cu-doped TiO_2_ nanoparticles. Int. Nano Lett..

[22] Pongwan P., Wetchakun K., Phanichphant S., Wetchakun N. (2016). Enhancement of visible-light photocatalytic activity of Cu-doped TiO_2_ nanoparticles. Res. Chem. Intermed..

[23] Geesi M.H., Ouerghi O., Elsanousi A., Kaiba A., Riadi Y. (2022). Ultrasound-Assisted Preparation of Cu-Doped TiO_2_ Nanoparticles as a Nanocatalyst for Sonochemical Synthesis of Pyridopyrimidines. Polycycl. Aromat. Compd..

[24] Zhu X., Wen G., Liu H., Han S., Chen S., Kong Q., Feng W. (2019). One-step hydrothermal synthesis and characterization of Cu-doped TiO_2_ nanoparticles/nanobucks/nanorods with enhanced photocatalytic performance under simulated solar light. J. Mater. Sci. Mater. Electron..

[25] Sunada K., Watanabe T., Hashimoto K. (2003). Studies on photokilling of bacteria on TiO_2_ thin film. J. Photochem. Photobiol. A Chem..

[26] Li G., Dimitrijevic N.M., Chen L., Rajh T., Gray K.A. (2008). Role of Surface/Interfacial Cu2+ Sites in the Photocatalytic Activity of Coupled CuO−TiO_2_ Nanocomposites. J. Phys. Chem. C.

[27] Irie H., Miura S., Kamiya K., Hashimoto K. (2008). Efficient visible light-sensitive photocatalysts: Grafting Cu(II) ions onto TiO_2_ and WO_3_ photocatalysts. Chem. Phys. Lett..

[28] Tamarani A., Zainul R., Dewata I. (2019). Preparation and characterization of XRD nano Cu-TiO_2_ using sol-gel method. J. Phys. Conf. Ser..

[29] Souvereyns B., Elen K., De Dobbelaere C., Kelchtermans A., Peys N., D’Haen J., Mertens M., Mullens S., Van den Rul H., Meynen V. (2013). Hydrothermal synthesis of a concentrated and stable dispersion of TiO_2_ nanoparticles. Chem. Eng. J..

[30] Sirichokthanasarp J., Trinh D.T.T., Channei D.A.D., Chansaenpak K., Khanitchaidecha W., Nakaruk A. (2020). Influence of Preparation Methods of TiO_2_ Nano-Particle on Photodegradation of Methylene Blue. Mater. Sci. Forum.

[31] Zhou X., Zhang X., Wang Y., Wu Z. (2021). 2D Graphene-TiO_2_ Composite and Its Photocatalytic Application in Water Pollutants. Front. Energy Res..

[32] Fernández-Catalá J., Sánchez-Rubio M., Navlani-García M., Berenguer-Murcia Á., Cazorla-Amorós D. (2020). Synthesis of TiO_2_/Nanozeolite Composites for Highly Efficient Photocatalytic Oxidation of Propene in the Gas Phase. ACS Omega.

[33] Tauc J., Grigorovici R., Vancu A. (1966). Optical Properties and Electronic Structure of Amorphous Germanium. Phys. Status Solidi B.

[34] Hanaor D.A.H., Sorrell C.C. (2011). Review of the anatase to rutile phase transformation. J. Mater. Sci..

[35] Shannon R.D. (1976). Revised effective ionic radii and systematic studies of interatomic distances in halides and chalcogenides. Acta Crystallogr. A Found. Adv..

[36] Nguyen Thi Thu T., Nguyen Thi N., Tran Quang V., Nguyen Hong K., Nguyen Minh T., Le Thi Hoai N. (2016). Synthesis, characterisation, and effect of pH on degradation of dyes of copper-doped TiO_2_. J. Exp. Nanosci..

[37] Krishnakumar V., Boobas S., Jayaprakash J., Rajaboopathi M., Han B., Louhi-Kultanen M. (2016). Effect of Cu doping on TiO_2_ nanoparticles and its photocatalytic activity under visible light. J. Mater. Sci. Mater. Electron..

[38] Wen L., Liu B., Zhao X., Nakata K., Murakami T., Fujishima A. (2012). Synthesis, Characterization, and Photocatalysis of Fe-Doped TiO_2_: A Combined Experimental and Theoretical Study. Int. J. Photoenergy.

[39] Moradi H., Eshaghi A., Hosseini S.R., Ghani K. (2016). Fabrication of Fe-doped TiO_2_ nanoparticles and investigation of photocatalytic decolorization of reactive red 198 under visible light irradiation. Ultrason. Sonochem..

[40] Zhang Z., Wang C.-C., Zakaria R., Ying J.Y. (1998). Role of Particle Size in Nanocrystalline TiO_2_-Based Photocatalysts. J. Phys. Chem. B.

[41] Qiu X., Miyauchi M., Sunada K., Minoshima M., Liu M., Lu Y., Li D., Shimodaira Y., Hosogi Y., Kuroda Y. (2012). Hybrid Cu_x_O/TiO_2_ Nanocomposites as Risk-Reduction Materials in Indoor Environments. ACS Nano.

[42] Gamage McEvoy J., Zhang Z. (2014). Antimicrobial and photocatalytic disinfection mechanisms in silver-modified photocatalysts under dark and light conditions. J. Photochem. Photobiol. C Photochem. Rev..

[43] Janczarek M., Endo M., Zhang D., Wang K., Kowalska E. (2018). Enhanced Photocatalytic and Antimicrobial Performance of Cuprous Oxide/Titania: The Effect of Titania Matrix. Materials.

[44] Applerot G., Lellouche J., Lipovsky A., Nitzan Y., Lubart R., Gedanken A., Banin E. (2012). Understanding the Antibacterial Mechanism of CuO Nanoparticles: Revealing the Route of Induced Oxidative Stress. Small.

[45] Othman S.H., Abd Salam N.R., Zainal N., Kadir Basha R., Talib R.A. (2014). Antimicrobial Activity of TiO_2_ Nanoparticle-Coated Film for Potential Food Packaging Applications. Int. J. Photoenergy.

[46] Meghana S., Kabra P., Chakraborty S., Padmavathy N. (2015). Understanding the pathway of antibacterial activity of copper oxide nanoparticles. RSC Adv..

[47] Ansari M.A., Albetran H.M., Alheshibri M.H., Timoumi A., Algarou N.A., Akhtar S., Slimani Y., Almessiere M.A., Alahmari F.S., Baykal A. (2020). Synthesis of Electrospun TiO_2_ Nanofibers and Characterization of Their Antibacterial and Antibiofilm Potential against Gram-Positive and Gram-Negative Bacteria. Antibiotics.

